# Hints and Improvements in the Practice of Medicine and Surgery

**Published:** 1799-04

**Authors:** 


### HINTS AND IMPROVEMENTS

### IN THE PRACTICE OF

### MEDICINE AND SURGERY.

### *Propofal for improving the Practice of Medicine, by the Introduction of Clinical Tables.*

IT is a remark, which cannot be too frequently and ferioufly repeated, that the greater number of errors and controverfies in medical practice, have arifen from the fuperficial and inaccurate mode of making *clinical obfervations.* If fuch were not deplorably the cafe, the medical world would not at prefent groan under a burthen of confufed defcriptions of difeafes, and, not unfrequently, mifreprefented facts, whether proceeding from the inadvertency or pathological ignorance of the obferver.

Much has lately been done in various parts of Europe, to remedy thefe effential defects, which have hitherto particularly difgraced the French and German fchools. While the former are amufing themfelves in framing new fyftems of pathology, founded upon the chemical combination of the elaftic fluids, or gafes, decompofed in the analyfis of animal fubftances; the latter have adopted the more Hippocratical method of examining the intricate labyrinth of difordered action in the human fyftem, by following the learned BACON's incomparable plan of judging from actual experience only, and proceeding from a fufficient number of particular facts, to form general conclufions, by way of *rational induction.*

From the correfponding accounts refpecting the prefent ftate of different medical fchools in Germany, we can with confidence affert, that the method of inquiry we allude to, efpecially prevails in the univerfities of *Jena, Goettingen, Vienna, Bamberg, Wurzburg,* &c. The late publications of DIETZ, GRUNER, GOTTHARD, and VOGEL, on *Symptomatology,* or the moft proper method of examining patients, and afcertaining the caufes of difeafe, have in this refpect, nearly accomplifhed a revolution. To gratify the curiofity of the Britifh reader, we have judged it equally neceffary and ufeful, to communicate a fpecimen of the Table now actually ufed, in moft of the hofpitals, as well as by many private practitioners in Germany. We fhall not attempt to point out the practical advantages of fuch Tables, if accurately attended to; as the utility of them, in reviewing the progrefs and fymptoms of the disease under treatment is so evident, that it can be doubted and vilified by those only, who are either passionately averse to all innovations, or who reject every improvement coming from others, and attended with a degree of trouble and attention.

---

### *Artificial Musk, in the Hooping-Cough.*

PROFESSOR HUFELAND, of Jena, an eminent and succesful practitioner, strongly recommends the use of musk, particularly that which is artificially prepared, in the epidemic hooping cough. He affirms, that this substance, after the proper emetics and resolvents had been premised, possesses the power of volatizing the more subtile acrimonious humours *, and expelling them through the pores of the skin. This remedy, although invented by the celebrated chemist, MARGGRAF, many years ago, and sanctioned by the great authorities of VAN SWIETEN, and STOELLER appears to be little, if at all, known or employed in this country. As it is a medicine comparatively cheap, and easily procured, we shall communicate to our readers the following approved method of preparing it.

Three drachms and a half of concentrated nitric acid are gradually dropped on one drachm of rectified oil of amber, which is previously poured into a wine glass. When this mixture is agitated, it grows hot, and emits offensive fumes, against the inhalation of which the operator must be on his guard. After having stood twenty-four hours, the compound acquires a resinous appearance; at the bottom of it we find a strongly acid fluid, but on the top of it a yellow resin, resembling musk in its fragrance. This resinous matter must be repeatedly washed, first in cold and then in hot water, until the acid taste be completely removed. Thus we obtain a substance which is equal in flavour, as well as in its medicinal properties, to the genuine natural musk, which is perfectly soluble in spirit of wine, which, like other resins, can be precipitated by water, and which always retains the scent acquired by this simple chemical process.

According to Dr. HUFELAND's experience, the artificial musk has been found of eminent service in the hooping-cough, as well as in all kinds of nervous diseases. Indeed, the oil of amber combined with the nitric acid, might lead us, *a priori*, to conclude their uncommon efficacy in nervous and

---

* We apprehend this assertion will excite a smile in those opponents of *humoral pathology*, who fondly ascribe *every* inordinate action in the system to some preternatural state of the nervous and muscular fibres. W.

and ſpaſmodic affections. As this ſubſtance is of a waxy and reſinous conſiſtence, it is moſt conveniently adminiſtered in the form of emulſions. For this purpoſe, from ten to twelve grains are triturated in a mortar, with a few almonds, and diluted with five or ſix ounces of water. Of this mixture, two tea-ſpoonsful are given every two hours, to a child from one to two years of age, and in a riſing proportion to older children. Many patients have received a cure from the uſe of this remedy, and a few occaſional emetics, without the aid of any other medicine. It generally produced a ſudorific effect, while it obviouſly diminiſhed and alleviated the fits of coughing; and not unfrequently it was attended with eruptions, which, in many inſtances, aſſumed the form of the true nettle-raſh; and by this favourable criſis, ſoon terminated the diſeaſe.

---

### *New Method of preventing Aſphyxia in Children.*

THE *Medico-chirurgical Journal* of Profeſſor TODE, Vol. III. No. III. publiſhed in German, at *Copenhagen*, announces an important diſcovery in the art of midwifery. M. HERHOLDT has found, on opening the bodies of animals ſtill-born, that the cavity of the tympanum is filled with the *liquor amnii* and phlegm (viſcous water); that this fluid is ejected after birth through the ſinus auditorius, and replaced by atmoſpheric air. This diſcovery has induced him to ſuppoſe, that the liquor amnii is likewiſe introduced into the trachea, and fills it ſo entirely, as to prevent the infant from breathing when born. Experiments made at the Veterinary School have confirmed this hypotheſis. Generally, Nature, by her own efforts, diſcharges this liquor; ſometimes, however, ſhe requires the aſſiſtance of art. The child cannot reſpire freely, till it be thus relieved. M. Herholdt is of opinion that this circumſtance produces more apparent deaths than is commonly imagined. It is not then enough to rinſe the throat of the child, but it ought to be placed in an attitude which facilitates the diſcharge of the liquor. The author has been ſo fortunate, in the courſe of laſt year, as to reſtore to life twelve out of thirteen children in this ſituation. Profeſſors ABILDGAARD and WIBORG have confirmed the experiment, by opening five puppies in embryo.

---

### *Medicines ſucceſsfully employed in Epilepſy.*

THIS formidable diſeaſe, againſt which innumerable remedies have hitherto been uſed, with little or no ſucceſs, ſeems at length, in a conſiderable variety of inſtances, to have yielded to medical treatment.

Much

Much as we deprecate the idea of curing diseases arising from *different* causes, by *similar* remedies, we should consider ourselves deficient in candour and justice, were we to withhold communications from the medical public, merely because they are not always derived from *professional* sources, or recommended by high authorities. Such is the case with respect to the first remedy we propose to describe.

There is, at present, a remedy much in vogue on the Continent, particularly in Germany, which is known by the name of '*Ragolo's Epileptic Powders.*' They have been lately analysed by physicians of the first eminence, and prescribed with uncommon success, in several very obstinate cases of epilepsy. This remedy was originally invented by *Meldola*, a Spaniard, who established its celebrity, by curing a Portuguese lady of high rank. In consequence of this event, the Court of Spain enjoined Dr. Ragola to try its effects; and, as this mercenary gentleman had not a sufficient share of liberality to make the composition of it known to the public at large, he sold it to a medical person in Germany, of an equally narrow mind, who is now carrying on with it a very lucrative traffic.*

According to the conjectures of CNOPE, who has chemically analysed Ragolo's powders, and communicated the result in *Baldinger's Magazine*, Vol. xi. p. 232, they consist of a mixture of valerian, orange-peel, sal ammoniac, and cajeput oil. Professor GMELIN, of Gottingen, gives a similar opinion in *Crell's Annals of Chemistry*, No. 12, for 1793; although it is supposed by others, that they are principally composed of the toad-stool (*lycoperdon bovista, Lin.*), valerian, and some other distilled oil; for it is well known, from thirty successful cases mentioned in the '*Universal Literary Gazette of Jena*,' No. 245, for 1793, that the lycoperdon operates almost as a specific in epileptic fits, particularly those arising from sudden dread or terror. The formula in which Drs. Baldinger and Gmelin have prescribed these powders, is nearly as follows: powder of the valerian root, half a drachm; magnesia alba, and sal ammoniac, each six grains; and cajeput oil three drops—to be taken for a dose. To secure the desired effect, we would recommend to add a grain or two of the dust of the lycoperdon, to each dose, and gradually to increase the quantity of the last article.

Dr.

---

* They are now sold by a Mr. Pfluger, of Nurenberg, in small boxes, at the enormous price of THREE GUINEAS each! Thus it appears that we are not *singular* in suffering a traffic to be carried on, which is rather encouraged than opposed, by those who ought to guard the unwary against fraudulent practices, instead of giving such impositions an apparent sanction by public and private documents. W.

Dr. THIELEMANN, a respectable practitioner in Germany, cured, by means of these powders, in the course of six weeks, a youth who had for three years been afflicted with epileptic fits: and Dr. DOLLE likewise, within a few weeks, restored to society a young woman, who for eighteen months, had been subject to attacks of epilepsy. Dr. JAHN, however, another German physician, affirms that he has found them of no service.

Dr. GESENIUS, of Nordhausen, observes in one of his latest publications, that in those cases of epilepsy which arise from acrimonious humours, and a preternatural degree of sensibility and irritability in the nervous system, he has successfully prescribed the misseltoe *(viscus albus, Lin.)* He administers it, as COLBATCH did, in the form of powders, three times a day, half a drachm each dose; at the same time he directs the patient to drink plentifully of an infusion made of four ounces of misseltoe, cut small; a handful of peony flowers; and two ounces of syrup of peony, or yellow roses.

---

From a letter of Dr. CAPPE, a promising young practitioner at York, addressed to Dr. DUNCAN, jun. of Edinburgh *, we extract the following facts relative to the internal use of the *nitrate of silver*. Dr. Cappe gave this medicine to a man who had been renewedly attacked with epilepsy soon after his marriage. The effects of it were greater than could reasonably have been expected; as the patient had suffered from the disease for more than twenty-five years. For a long time there had not been an interval of more than ten days between his fits. He began to use the medicine on the day after he had many fits. He took a quarter of a grain thrice a day, and had no fit for a month. After this he enjoyed an interval of five weeks; and though the intervals became gradually longer, Dr. Cappe could not ascertain that his fits were less violent.

In the same letter, Dr. Cappe takes notice of a paper read by Dr. SIMS before the Medical Society of London, on the use of the *nitrate of silver in epilepsy*, in which paper Dr. Sims mentions, that it sometimes aggravated the disease at first, though it afterwards effected a complete cure. Dr. Cappe remarks, however, that he had not observed such an effect; though he had seen this substance used, particularly at the Carey-street Dispensary, in London, in many epileptic cases, with more or less advantage.

After having informed us, that he had also prescribed the nitrate of silver in one case of *angina pectoris*, in which it removed all the symptoms; in two

* *Dr. Duncan's Annals of Medicine* for 1798, p. 456, and following.

two cases of *dyspnœa,* where it had a powerful and speedy effect; and in one case of *hysteria,* with some apparent benefit. Dr. Cappe accompanies these facts with the following useful remarks: "This medicine, in general, keeps the bowels open; it sometimes produces diarrhœa, but it has not occasioned any painful effect in the cases under my observation, though given in the quantity of half a grain, three times a day. The effect of it (proceeds Dr. Cappe), in the case I have mentioned, cannot, I think, be attributed to its aperient operation: for, in the case of angina pectoris, it was frequently necessary to give aloetic pills, to prevent costiveness. No other remedies were employed with the nitrate of silver, except in the case of hysteria. In this instance it was necessary to unite opium with it, to prevent its purgative effects."

To conclude these important accounts relating to a disease of so obstinate and frequently complicated a nature, we shall only observe, that it appears to us of the first consequence to discover, if possible, the proximate and predisposing causes of epilepsy. The latter are perhaps so hidden in the pathology of the human mind and body, as not to be easily traced to their true source and origin: but the proximate causes have certainly not been hitherto sufficiently attended to. It is well known to experienced practitioners, that the epileptic convulsions, so common in early youth and infancy, not unfrequently proceed from some material stimulus, such as worms in the intestines, and particularly the *tape-worm,* without accounting for it from preternatural irritability, or indirect debility of the muscular or nervous fibres. Several ingenious methods of discovering this worm, which plays so decided a part in the diseases of children, have lately been devised, and not altogether without success. Dr. Sachse, of Parchim, in the dutchy of Mecklenburg, has observed, that a patient under his treatment for the tape-worm, regularly discharged long pieces of it in the morning, after she had eaten a small quantity of strawberries at night. This lady remarked the same phenomenon to recur a twelve month after she had first taken notice of this singular fact, which seems to claim the attention of practitioners. There is however nothing mysterious or extraordinary in this phenomenon. Strawberries, like all other vegetable substances which contain much water*, evolve large quantities of fixed air, or carbonic acid gas, in the bowels of animals; and, as this species of gas is of a highly deleterious and irrespirable nature, we need not wonder,

* While I was writing this article, a gentleman of sufficient respectability to vouch for the truth of the following fact, informed me, that a poor woman at Islington had preserved two long pieces of a tape-worm, the whole of which was expelled by the use of a simple decoction of carrots in water, which she had drank, for a considerable length of time, in large quantities. W.

wonder, that the eating of ſtrawberries produced this uncommon effect. The want of ſtrawberries, in this inſtance, might be very effectually ſupplied by employing the following mixtures: diſſolve two drachms of ſalt of tartar in twelve ounces of water, and dilute four ounces of the acid of lemons in twenty ounces of water; each ſolution to be kept in ſeparate bottles. Of the former mixture, the patient takes firſt half a tea-cupful, and immediately after it a whole tea-cupful of the latter. Theſe draughts ſhould be repeated every hour or two, till the tape-worm moves: now it becomes neceſſary to adminiſter ſtrong cathartics, ſuch as either jalap, ſcammonium, or aloes, with a proportionate addition of calomel, to accelerate the operation. This method will in general, be found more certain in expelling that harraſſing worm than any of the ſpecifies, not even the infuſion of carrots, or the powder of the male fern-root, excepted.

The ingenious and ſuccefsful experiments lately made by Dr. FRICKE, of Brunſwick, to diſcover and deſtroy the tape-worm by means of electricity, we are obliged, for want of room, to defer to our next Number.

---

### *On the Treatment of inveterate Ulcers.*

THE frequent failure in the attempt of curing ulcers, of the lower extremities in particular, has by many been conſidered as an opprobrium to the faculty. Thoſe who are unacquainted with the laws of the animal economy, as well as with the difficulties which medical practitioners experience, in aſcertaining the origin or cauſe of diſeaſes among patients of a certain deſcription, will not paſs ſo haſty a judgment.

About ſix years ago, Mr. FRAHM, a ſurgeon in the Daniſh dominions, publiſhed a treatiſe in which he recommended the cure of old ulcers of the legs, by exciting an artificial inflammation round the parts affected, by means of turpentine or other ſtimulating plaſters, keeping thoſe parts properly ſecured by bandages, and the ulcers regularly dreſſed with ſimple ointments, till nature, with the aſſiſtance of the neceſſary internal remedies, according to the circumſtances of the caſe, recovers ſufficient tone and ſtrength to heal the enlarged ulcers. Another method equally rational has been lately ſuggeſted by Mr. BAYNTON, of Briſtol, in his "*Deſcriptive Account of a new Method of treating old Ulcers of the Legs.*" 8vo. Briſtol, 1797. He aſſerts that the means propoſed by him will, in moſt inſtances, be found ſufficient to accompliſh cures in the worſt caſes, without pain or confinement. After having been repeatedly diſappointed in the cure of old ulcers, Mr. Baynton determined on *bringing the edges of ſuch ulcers nearer together by means of*

*ſlips*

*flips of adhefive plafters.* To this he was chiefly led, from having frequently obferved, that the probability of an ulcer continuing found, depended much on the fize of the cicatrix which remained after the cure appeared to be accomplifhed; and from well knowing, that the true fkin was a much more fubftantial fupport and defence, as well as a better covering, than the frail one which is obtained by the affiftance of art. But, when he had recourfe to the adhefive plafter, with a view to leffen the probability of thofe ulcers breaking out again, he little expected, that an application fo fimple would prove the eafieft, moft efficacious, and moft agreeable means of treating ulcers.

Although the firft cafes, in which Mr. Baynton tried this practice, were of an unfavourable nature, yet he had foon the fatisfaction to perceive that it occafioned very little pain, and materially accelerated the cure, while the fize of the cicatrices were much lefs than they would have been, had the cures been obtained by any of the common methods.

At firft, however, the fuccefs was not quite perfect; as in many inftances he was not able to remove the flips of plafter, without removing fome portion of the adjacent fkin, which, by occafioning a new wound, proved a difagreeable circumftance, in a part fo difpofed to inflame and ulcerate, as in the vicinity of an old fore. He therefore endeavoured to obviate that inconvenience by keeping the plafters and bandages well moiftened with fpring-water, for fome time, before they were removed from the limb. He had foon the fatisfaction to obferve, that the inconvenience was not only prevented, but that every fucceeding cafe juftified the confidence he now began to place in the remedy. He alfo difcovered, that moiftening the bandages was attended with advantages which he did not expect: while the parts were wet and cool, the patients were much more comfortable in their fenfations, and the furrounding inflammation was fooner removed than he had before obferved it to be.

By the mode of treatment here recommended, Mr. Baynton found, that the difcharge was leffened, the offenfive fmell removed, and the pain abated in a very fhort time. But, befides thefe advantages, he alfo found, that the callous edges were in a few days level with the furface of the fore; that the growth of fungus was prevented, and the neceffity of applying painful efcharotics much leffened, if not entirely done away. Mr. Baynton very properly gives a minute defcription of the method which he would recommend, of treating fuch ulcers in their different ftages; but, for thefe ufeful particulars, we refer the reader to the abovementioned treatife.

Among the internal remedies that have been lately propofed, and often employed with fuccefs by phyficians on the Continent, is the extract of the wild wild hyſop *(gratiola officinalis, Linn.)*; by which, according to the account given by Dr. Wendt*, four perſons have been cured of inveterate ulcers of the legs, attended with a diſcharge of ichorus matter. Dr. Wendt, however, adds, that patients ſubject to hermorrhages, particularly women during menſtruation, ought, under theſe circumſtances, to abſtain from the uſe of this extract; and others of a cachectic diſpoſition, which is generally prevalent in theſe complaints, ſhould take it at night only; for, if uſed in the morning, it is apt to occaſion violent purging and vomiting. The moſt proper method of adminiſtering it, is that of diluting two drachms of the extract of gratiola (which is eaſily prepared by inſpiſſating a ſtrong decoction of the flowers of the hyſop to the conſiſtence of a thick ſyrup) with four ounces of water, and directing the patient to take at firſt one table-ſpoonful at night, to increaſe the doſe gradually to two table-ſpoonfuls; and then to begin likewiſe to take a ſpoonful of this draught every morning. In that ſtage of pulmonary conſumption which ariſes from the abſorption of pus from large ſuppurating ulcers, and the ſubſequent hectic fever, it is confidently affirmed, that the prudent uſe of the extract of gratiola has frequently afforded unexpected relief, when there was was little hope of the patient's recovery.

Although we are no advocates for the application of external remedies in caſes of inveterate ulcers, being firmly perſuaded, that the nature of malignant ſores cannot be changed by external applications alone, yet we may be allowed here to ſpeak from our own experience, that a weak ſolution of the *nitrate of ſilver*, in the proportion of one grain to two ounces of water, is perhaps the moſt efficacious lotion that can be recommended with ſafety, to correct and check the diſcharge of fetid and putrid ſanies from deep-ſeated ulcers.

Another external remedy in ulcerations of the legs we learn from a late account communicated by Dr. Filter to Dr. Gesenius, who has inſerted it in his excellent "*Manual of Therapeutics*," 2nd edition, 8vo. Stendal, 1796, in German: and as this remarkable fact is as yet little known in this country, we ſhall here give a tranſlation of Dr. Filter's report, nearly in his own words:—"I have (ſays this excellent phyſician), in two caſes of inveterate ulcers of the legs, made uſe of the *Anguſtura bark*. The firſt caſe was that of a lady who had long paſſed the period of menſtruation, and, for many years, had been afflicted with arthritic pains. The ulcer, which had continued open for ſeveral months, and began to extend rapidly, was completely cured

* Vide his "Accounts of the Progreſs of the Clinical Inſtitution at Erlang." Number VI. publiſhed about the year 1794.

cured by means of frequent fomentation, with a decoction of this bark. In a similar case of a woman, whose menstruation was suppressed, I directed to dust the fetid, ichorous ulcer on the lower part of the thigh with the powder of Angustura bark, which not only produced more favourable pus in the course of a few days, but likewise performed a complete cure within a few weeks; without having prescribed, either in this, or in the preceding case, *any internal remedies*, which might have had a particular effect in contributing to the speedy cure of the patients."

To conclude this article, we shall take notice of a work which appeared some time ago on the treatment of ulcers, but which does not mention either the use of Gratiola or Angustura bark; though, in other respects, it is replete with useful practical remarks, and ingenious theoretical speculations; viz. "*A Prize Essay on the best method of treating inveterate ulcers of the lower extremities:* by F. X. Mezler, 4to. Vienna, 1792. In a future number we propose to furnish our readers with a concise abstract of this classical treatise.

---

### *Venereal Chancres cured by Alkalies.*

Dr. Mitchill, of New-York, informs us, in the American "*Medical Repository*," Vol. II. Numb. II. that venereal ulcerations have been removed with surprising quickness, by the local application of the common vegetable fixed alkali, or the carbonate of pot-ash. A successful and conclusive experiment has been made by that ingenious Professor, which (in his own words) "may serve to confirm the doubts of such gentlemen who disbelieve the wonders said to be wrought by the nitric acid, and, at the same time, to give new matter of reflection to those who have ascribed to it something like a specific mode of action.—A gentleman, a few days after impure coition, felt an itching on the præputium, which soon became ulcerated. As soon as the chancre was discovered, it was cauterized with the lunar caustic, and dressed with ointment strongly impregnated with the red precipitate of mercury.

"Notwithstanding repeated dressings, and the strictest observance of cleanliness in all respects, the ulcer continued to spread, and had invaded a considerable space on the prepuce. Finding it yield in no degree to these local applications, recourse was had to mercurial frictions, which were performed by rubbing the blue ointment in considerable quantities on the legs and thighs. Before a salivation came on, the appearance of the ulcer mended, and by degrees it healed. A few days after, the same gentleman having having repeated his impure careſſes, was troubled with a new chancre, cauſed, apparently, from freſh poiſon breaking out near the frænum. Immediately on diſcovering it, while yet exceedingly ſmall, and not larger than to contain a pin's head, it was touched with lunar cauſtic, and dreſſed with precipitate ointment, as the former had been; and in a conſtitution now charged with mercury, it was expected a cure would be very eaſy and expeditious. This, however, was not the caſe. The chancre continued to enlarge, to inflame, and to become more and more troubleſome, inſomuch, that it was apprehended, there would be a neceſſity for repeating the mercurial friction.

" But before recourſe was had again to this tedious and troubleſome remedy, it occurred to me, from the *analogy of volatile alkali in curing bites of venemous ſerpents, and of the fixed alkali and lime, in overcoming thoſe acid products of putrefaction, which poiſon the atmoſphere, and induce epidemic diſtempers,* that it was worth the while to try the effects of an *alkaline application,* to ſubdue the *virus of ſyphilis:* accordingly, the ulcerated ſurface was covered with ſome deliqueſcent ſalt of tartar. On being firſt applied, it excited ſevere ſmarting, which, however, laſted but a ſhort time. It was dreſſed with lint, and on examining it ſome hours afterwards, it looked better, and appeared to ſpread no more; after a ſecond application of pot-aſh, the appearance of the ulcer was apparently changed for the better; on a third application, it dried up, healed intirely, and has been well ever ſince.

" Whether, in this caſe, the pot-aſh operated upon the ſolids, and introduced in them a new action, incompatible with the morbid action, or whether it produced its effects by neutralizing the contagious matter on the ſurface of the ſore, are queſtions which the phyſioligiſt may conſider and explain at his leiſure. This experiment ſhows, at leaſt, that there is nothing ſpecific in the action, either of mercury or of nitrous acid, on venereal ulcers. Abundance of inſtances of chancres cured by alkali, have ſince occurred in the New-York Hoſpital.

---

### *Subſtitutes propoſed for the Peruvian Bark, in Intermittents.*

OF the almoſt innumerable remedies which have, from time to time, been introduced and again relinquiſhed, in the cure of intermittents, the bark is the only one which has now, for upwards of a century, maintained its well-merited reputation. It cannot, however, be denied that there are many other valuable valuable substances in nature, which, if they were properly and cautiously employed, would answer a similar purpose, and prevent the necessity of sending for this expensive foreign drug, as well as the frequent abuses to which it is now exposed, from its having among the vulgar acquired the character of a specific in agues, and from its indiscreet and promiscuous administration in every case of intermittent fevers, and to every patient, even although it appear to agree with the stomach. That much mischief may be and is daily done, in this respect, we shall endeavour clearly to prove in a future essay on this interesting subject. Let it suffice, at present, to point out those remedies which, since the time that Dr. FOWLER first recommended solutions of arsenic in *regular* practice, have been employed with success by other respectable practitioners.

The first and most conspicuous of these, the avens root (*geum urbanum Linn.* which according to the experiment of Dr. VOGEL (resident physician at Ratzeburg in Germany, physician to his Britannic Majesty, and son of the great nosologist), has lately cured three quartan agues, by directing half a drachm of the powder of this root to be taken, every hour through the day, and half a drachm of the Peruvian bark, with six grains of calomel in the morning. Thus, the most obstinate quartans, which had resisted the united virtues of the bark and other powerful febrifuges, yielded to the operation of the geum and calomel, *without exciting salivation.* It further deserves to be remarked, that the geum urbanum had long been employed by the country people in the vicinity of Eksjoe, in Denmark, before its effects were ascertained in regular practice.

The frequent and successful experiments made with this root, place the efficacy of it, in intermittents, beyond a shadow of doubt. It possesses great antiseptic and astringent powers, and at the same time moderates irritation; hence it proves so excellent a febrifuge. It may be used either in a spirituous infusion, by macerating eight ounces of the powdered root in sixteen ounces of proof-spirits, for two or three days, and directing an ounce of this infusion to be taken three or four times a day, in cold water, during the intermission of the fever; or in a decoction, by boiling one ounce of the dry root in 24 ounces of water down to sixteen ounces, and causing the patient to take two ounces of it, in the same manner as the preceding; or lastly, and with a greater prospect of success, the powder of the geum may be taken in substance, in repeated doses, as has already been stated. In this way, it is a safe and effectual remedy against the most obstinate agues, and well deserves a fair trial, in many other inveterate diseases, arising from inordinate action in the sanguiferous and nervous systems. The thick root, however, especially when collected in spring and autumn, is much preferable to the thin fibrous parts, which are so weak that they scarcely possess any virtue. The uncommon effects of this medicine medicine ſtand further confirmed by the evidences of two other phyſicians at Kiel in Holſtein, Drs. WEBER and KOCH. They have ſucceſsfully employed it in no leſs than two hundred caſes of intermittents, and their teſtimony is confirmed by MURRAY, in his "*Apparatus Medicaminum,*" vol. iii. who recommends much larger doſes—but the experience of other phyſicians is rather leſs favourable to the character of this extraordinary root.

The next remedy we ſhall mention, is that of the *Datiſca canniba* of Linnæus. This plant* has lately been exhibited, by a conſiderable number of Italian phyſicians, with an unprecedented degree of ſucceſs. We ſhould not have ventured to number the datiſca among the ſubſtitutes for bark, had we not before us the authority of JOHN MARSIGLY, the preſent Profeſſor of Botany in the Univerſity of Padua, who inform us, that it will ſtand the ſevereſt froſt, and requires no particular care; for the ſtem withers away in autumn, and the root again rapidly ſhoots new ſtalks in ſpring. It flowers about the end of May, and if ſeveral plants of both ſexes have been ſet near each other, they produce ſeeds which become ripe in Auguſt.

The ſtrong bitter taſte of this vegetable led the Profeſſor to ſuppoſe, that it poſſeſſed great antiſeptic and febrifuge virtues; hence he tried it, but with precaution, in a variety of caſes. The ſucceſs he met with far exceeded his expectations: and the ſubſequent trials made by one of his pupils, Dr. QUATTERI, Profeſſor of Botany in Parma, and by Dr. PINCETTI, Profeſſor of Surgery and Midwifery in the ſame Univerſity, fully confirmed his own conjectures and experiments. Similar accounts of the excellence of the datiſca in intermittents have been tranſmitted to Prof. Marſigly, by Dr. ZULATTI, of Florence, Dr. PONTELLI, of Udina, and Dr. DE ROSSI, of Oſſaro, the laſt of whom goes ſo far as to aſſert, that he directed a decoction of the leaves to be taken three ſucceeding mornings, in the quantity of four ounces each morning (not ſtating, however, whether in one, or ſeveral divided doſes); and that he found this decoction to be attended with the moſt favourable effects, *in all periodical fevers.*

Marſigli has employed the freſh and dried leaves, as well as the root of the datiſca, in infuſions, decoctions, and powders, and the ſucceſs was uniformly

* For the botanical character of the *datisca* we refer the reader to the work of PROSPER ALPINUS, '*De plantis exoticis,*' 301. Tab. 300, and 298, where he will find a deſcription of it under the reſpective names of *cannabis lutea sterilis,* and *cannabis lutea fertilis Contareni.*—VESLING, the ſucceſſor of Alpinus, and MORISON, preſerved theſe names; though TURNEFORT called it, *cannabina Cretica florifera & fructifera.* It is not an eaſy conjecture, why Linnæus terms it the *datisca cannabina.*

uniformly favourable. He also tried the bitter seeds of this plant reduced to fine powder, the result of which he sent to Dr. Minconi, resident physician at the Bath of Abano, with a further view to ascertain its medicinal properties. That practitioner gives the most satisfactory account of the antifebrile virtues of this powder, which he observed in four different cases of obstinate agues, after bark and many other medicines had been used with no effect; here he directed twelve grains to be taken for a dose in the mornings. The first patient, in the fourth month of pregnancy, and during the whole time she was afflicted with the ague, obtained relief from one dose, which she took on the day of intermission, and vomited a yellow viscid matter. On the day following, indeed, the fever returned, but for the last time. The second patient was a woman thirty-four years of age, whose fever disappeared on taking a similar dose of twelve grains, and after many critical evacuations by stool. The third was a boy ten years of age, much reduced, of a yellow complexion, impaired digestion, no appetite, and a very swollen belly. He likewise took twelve grains of the powder, vomited twice a quantity of viscid, greenish matter, was liberated from the fever, his appetite and strength returned, and he has now, for three months, enjoyed the most perfect state of health. The fourth patient was a middle-aged woman, extremely reduced and emaciated by a tertian ague, without the least appetite, and threatened with pulmonary consumption. After taking one usual dose of this powder, the fever never returned, and she is gradually recovering.

Professor Marsigli very prudently avoids to account for the mode in which this plant operates on the human constitution, as we have not arrived at any such certainty, in respect of the Peruvian bark and other medicines considered as limited specifics. But besides the incomparably lower price of the datisca, it has another great advantage, that it may always be procured fresh, and in an unadulterated state; that it does not, like the bark, induce oppression of the stomach, obstipation, &c.; that the first dose is generally sufficient to cure the ague, so that the second and third seldom become necessary; and lastly, that the paroxysms of the fever suppressed by it rarely, if ever, return.

The immediate effect of this plant is manifestly on the organs of digestion, the intestinal canal, and the kidneys; hence it is an object to which the practitioner ought to direct his principal attention, to take an accurate and comprehensive view of the particular constitution of his patient.

From the evacuating, diuretic, and alterative powers of the datisca, we may reasonably expect to derive considerable benefit in those diseases, where

ſuch changes are indicated.—Although the root is the bittereſt part of this plant, it is not the moſt active; as the leſs bitter ſeeds are far preferable.

The next ſubſtitute for the bark in agues, in point of efficacy and celebrity, is the bark of the willow, of which we ſhall mention ſix ſpecies, together with their reſpective virtues, according to the *comparative experiments* made with them and the Peruvian bark, particularly by Dr. GUNZ, an accurate chemiſt in Germany. The *ſalix alba* contains as many volatile and gummy parts as the red bark, but leſs than the common pale bark; on the contrary, it contains more reſinous and leſs earthy particles than either: in the *ſalix pertendra*, the proportion of the former is greater, that of the latter ſmaller, than in both ſpecies of the Peruvian; the *ſalix fragilis* has more volatile parts than the red, and leſs than the common bark, but leſs earthy, and more gummy and reſinous parts than either; the *ſalix caprea* exhibits more reſinous and leſs earthy parts than either; but, as many gummy as the red, and leſs of theſe than the common bark; the *ſalix vitellina* yields more reſin and leſs earth than either, the ſame quantity of gum as the common, and more than the red bark; the *ſalix amygdalina*, more gum and reſin, but leſs earth than either, more volatile parts than the common, and not leſs than the red bark.

In ſuch caſes, therefore, where, according to the doctrines of humoral pathology, the fluids require to be inſpiſſated, the bile improved, and the vital powers raiſed, the bark of the ſalix vitellina and alba is, according to the experience of Dr. Gunz, much preferable to the other ſpecies. The ſalix fragilis he particularly recommends for external applications; and of the ſame opinion is Dr. LOFFLER, in "RICHTER's *Chirurgical Library*," Vol. VII. p. 789, where this ſpecies of bark is uniformly recommended in preference to the Peruvian. The ſame phyſician has, with great advantage, employed this ſubſtance, in the year 1772, in a malignant epidemic fever of a putrid form, then prevailing.—In hemorrhages, he obſerved all kinds of the willow bark to produce ſalutary effects. In caſes of nervous debility, induced by exceſſive indulgence in venery, he found the ſalix vitellina the moſt efficacious; in involuntary diſcharges of ſemen, fluor albus, and againſt worms, the ſalix amygdalina was the moſt powerful; when adminiſtered in the form of extract in the two firſt diſeaſes, and in ſubſtance as an excellent anthelmintic.

Laſtly, the *bark of mahogany*, in powder, has been ſtrongly recommended on the authority of Dr. LIND, of Jamaica, as a valuable febrifuge, which has likewiſe been occaſionally preſcribed by phyſicians in this country with pretty general ſucceſs. Dr. MICHAELIS, of Leipzig; Profeſſor LODER, of of Jena; and Mr. BUCHHOLZ, a respectable mineralogist, all have, in their several writings, confirmed the remarkable antifebrile virtues of this cheap substance, when used in powder, and in the same proportions as the Peruvian bark.

---

### *A singular Case of Dropsy, after the Repulsion of the Itch, cured by Sulphur.*

FROM an account communicated by a physician of Worms, in Germany, to Professor LODER, of Jena, the editor of "*The Journal of Surgery, Midwifery, and Medical Jurisprudence*," Vol. II. Numb. I. we learn the following curious fact, which has been further corroborated by the experience of Professor HUFELAND, who considers this case as conveying a decisive confirmation of the truth contained in the old doctrine of the *metastases*, or the translation of morbid humours from one part of the body to another.

A strong lusty woman, who had long dabbled in various quack medicines reputed among the vulgar as specifics in dropsy, was at length obliged to submit to the operation of paracentesis, which had been frequently repeated, with no lasting success. As she had formerly been troubled with the itch, which was cured by the use of sulphurated ointments, she was directed to take sulphur internally. The itch again made its appearance, and an inflamed tumour, of the size of a dollar, began to settle in the region of the navel. This tumour at last burst, and evacuated no less than forty eight pints (*schoppen*, in Germany,) of a fetid and putrid sanies. Several such evacuations succeeded, so that the whole of them is stated to amount to the enormous and almost incredible number of 484 pints!!! The itch at the same time, became violent, and the patient gradually recovered.—Gentle reader! it is certainly invidious to *doubt* facts, but sometimes equally difficult to *believe* them.

---

Mr. KAUFER, a respectable surgeon of Naugardt, in Pomerania, having observed that tubercles of the lungs generally arose from arthritic matter, and that sulphurated waters have frequently been administered with remarkable success in the *gout*, has lately made the following ingenious proposal. He advises cousumptive patients, whose lungs are supposed to be affected with tubercles, constantly to breathe the atmosphere of such rooms, particularly in hospitals, as are inhabited by no other but psoric patients, or those who use *sulphur* internally; so that the vapours arising from the decomposition of that substance in the animal body may have immediate access to the lungs.---We sincerely wish, that this apparently well-founded conjecture may not be lost to the medical world; as sulphur cannot be given to phthisical patients with safety, by the organs of digestion.

